# Bioprinting of GelMA/PEGDA Hybrid Bioinks for SH‐SY5Y Cell Encapsulation: Role of Molecular Weight and Concentration

**DOI:** 10.1002/mabi.202400587

**Published:** 2025-03-25

**Authors:** Hexin Yue, Yaxin Wang, Samantha Fernandes, Cian Vyas, Paulo Bartolo

**Affiliations:** ^1^ Department of Mechanical Aerospace and Civil Engineering University of Manchester Manchester M13 9PL UK; ^2^ Singapore Centre for 3D Printing School of Mechanical and Aerospace Engineering Nanyang Technological University Singapore 639798 Singapore

**Keywords:** bioprinting, GelMA, PEGDA, peripheral nerve regeneration, SH‐SY5Y cells

## Abstract

Current clinical interventions for large peripheral nerve gap injuries are limited. Bioprinting provides opportunities to develop tissue engineered constructs that provide a biomimetic environment to guide nerve regeneration. However, hydrogels that are cell‐instructive, mechanically compliant, and have an appropriate biodegradation profile for nerve guidance conduit applications are limited. In this study, a photocrosslinkable gelatin methacryloyl (GelMA) and polyethylene glycol diacrylate (PEGDA) hybrid bioink is developed. The role of PEGDA molecular weight and concentration in tuning the hydrogel physicochemical and biological properties is evaluated. PEGDA modulated the hydrogel network structure and properties in a molecular weight and concentration dependent manner. A lower molecular weight and high concentration induced high crosslinking density thus improving compressive modulus, lower swelling, and a slower degradation profile. The bioinks showed good printability and are able to fabricate multi‐layer constructs with high shape fidelity and flexibility. The SH‐SY5Y cells maintained high cell viability after bioprinting in all bioinks. However, cells showed limited metabolic activity and spreading in the GelMA/PEGDA hydrogels with both high concentration and molecular weight. This preliminary study provides guidance on the use of specific molecular weights and concentrations in GelMA/PEGDA bioinks for the bioprinting of SH‐SY5Y cells.

## Graphical abstract

Bioprinting offers a promising approach for repairing large peripheral nerve gap injuries, but suitable hydrogels remain limited. This study developed a visible light photocrosslinkable GelMA/PEGDA hybrid bioink and investigated how PEGDA molecular weight and concentration affect hydrogel properties. Higher crosslinking density improved mechanical strength and stability but reduced cell activity. Findings provide insights for optimizing GelMA/PEGDA bioinks for nerve regeneration.

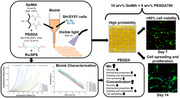

## 1 Introduction

Peripheral nerve injury (PNI) is caused by trauma or disease and is a significant clinical burden with more than 5 million new cases reported globally each year.^[^ ^1^ ^]^ Nerve regeneration can spontaneously occur in nerve gaps as small as a few millimeters. For larger nerve gaps, axons must regenerate at 1–3 mm per day to reach the distal motor endplate and may not fully recover.^[^ ^2^ ^]^ This results in pain, sensorial complications, and mobility issues in patients. Clinical treatment mainly focuses on nerve suturing and autologous nerve grafting techniques.^[^ ^1^, ^3^ ^]^ However, nerve sutures require precise surgery and may result in excessive tension, and autografts have limited availability and morbidity at the donor site. Commercially available nerve guidance conduits (NGCs) are ineffective for gaps over 30 mm and underperform compared to autografts for smaller gaps.^[^ ^4^ ^]^ Although there have been significant advances related to the development of biocompatible and biodegradable materials, directing axonal outgrowth, supporting glial cell proliferation and migration, and biomechanically compliant structures, this has currently not resulted in clinical translation of advanced NGC. The development of tissue engineered NGCs and bioprinting shows potential to modulate the physicochemical and biochemical 3D microenvironment to mimic the extracellular matrix (ECM), thus promoting cell regeneration and offering a potential route to commercialization.^[^ ^5^ ^]^ Biomaterials commonly used for the development of NGCs include collagen, chitosan, silk, gelatin methacrylate (GelMA), hyaluronic acid (HA), and poly(ethylene glycol) diacrylate (PEGDA).^[^ ^6^, ^7^, ^8^, ^9^ ^]^

Bioprinting, a computer‐aided process that enables precise fabrication of complex and multi‐material 3D cell‐laden constructs, is a promising strategy in tissue engineering and regenerative medicine applications.^[^ ^10^ ^]^ A range of bioinks, cell‐based formulations, have been introduced to create an ideal microenvironment.^[^ ^11^ ^]^ The specific biochemical and biophysical properties of the ECM guide cell behavior. Thus, a biomimetic microenvironment similar to the target tissue can be engineered through the precise selection of biomaterials, crosslinking strategies, and the inclusion of biostimulatory factors. Bioprinting has been utilizedin the development of NGCs, however, the development of cell‐laden NGC specific bioinks is limited.^[^ ^5^ ^]^ Balancing printability and cytocompatibility whilst providing cell instructive properties is a significant challenge.^[^ ^11^ ^]^ For example, Wu et al.^[^ ^12^ ^]^ have successfully bioprinted a gelatin/alginate bioink containing rat Schwann cells with high cell viability and expression of neural specific markers. However, significant advancements are required in selecting the optimal cells, providing a biomimetic environment, and matching the regeneration rate to enable a clinically efficacious bioprinted NGC. Subsequently, the engineering of materials to facilitate this is of utmost importance.

Gelatin has attracted significant attention for use in bioinks due to its natural origin (collagen derived), biocompatibility, cell binding sites and affinity, and biodegradability. Gelatin bioinks have been shown to be capable of aiding the specific delivery of cells in multi‐layer liver fibrosis‐on‐a‐chip^[^ ^13^ ^]^ or as a rheological modifier to enable the bioprinting of a variety of biomaterials.^[^ ^14^ ^]^ However, gelatin is typically modified, for example, with methacrylic anhydride (MA) to form GelMA to enable rapid photocrosslinking using mild conditions to form a physiological temperature stable and cytocompatible hydrogel.^[^ ^15^ ^]^ The biophysical and biochemical properties of GelMA hydrogels can be adjusted by controlling the type of gelatin used, the degree of functionalization, concentration, and processing parameters (e.g., bioprinting photocrosslinking conditions).^[^ ^15^, ^16^, ^17^ ^]^ Thus, enabling tailorable porosity, swelling and degradation profiles, mechanical properties, and cell behavior. Furthermore, GelMA has room‐temperature printability, allowing the fabrication of multi‐layer and multi‐cellular constructs, hence, has been widely used as a bioink.^[^ ^18^ ^]^

However, GelMA has poor mechanical properties and lacks long‐term stability to provide a robust structure for PNI applications.^[^ ^7^, ^19^ ^]^ Therefore, combining GelMA with PEGDA to create a hybrid bioink with tuneable mechanical, swelling, and degradation properties is a promising option for nerve regeneration applications. PEGDA hydrogels are cytocompatible, non‐immunogenic, and have slow in vitro and in vivo biodegradation profiles.^[^ ^20^, ^21^ ^]^ Furthermore, different molecular weights and polymer concentrations can modulate the internal structure of PEGDA and hybrid hydrogel networks, fundamentally affecting the biophysical properties and cell response.^[^ ^22^, ^23^ ^]^ GelMA/PEGDA composite hydrogels have been shown to tailor the mechanical, biodegradation, diffusion, and swelling properties of GelMA by adjusting the concentration of PEGDA.^[^ ^18^ ^]^ Therefore, a GelMA and PEGDA hybrid hydrogel has the potential to match the regeneration rate of peripheral nerve tissue. Although composite systems of PEGDA, GelMA, and other materials have been developed, the effect of molecular weight and polymer concentration of PEGDA on the hydrogel properties and their potential in peripheral nerve tissue engineering applications is unclear.^[^ ^7^, ^20^, ^23^, ^24^, ^25^, ^26^, ^27^, ^28^ ^]^

This preliminary study explores the role of PEGDA molecular weight and concentration in tuning the properties of a hybrid covalently crosslinked GelMA/PEGDA bioink. The influence on the hydrogel microstructure, stiffness, degradation, rheology, printability, and biocompatibility are investigated. Visible light photopolymerisation bioprinting was used to fabricate stable multi‐layer structures encapsulating neuroblastoma SH‐SY5Y cells. The bioinks developed in this study showed tuneable physical properties, good printability, and cytocompatibility with potential for usage in peripheral nerve tissue engineering applications.

## 2 Experimental Section

### 2.1 Materials

Gelatin (gel strength 300, type A, porcine skin), methacrylic anhydride (MA, 94%), poly (ethylene glycol) diacrylate (PEGDA, Mn = 700, 2000 and 4000), ruthenium powder (ris(2,2′‐bipyridyl) dichlororuthenium(II) hexahydrate (Ru), sodium persulfate powder (SPS), deuterium oxide (D_2_O) containing 0.05 wt.% 3‐(trimethylsilyl)propionic‐2,2,3,3‐d4 acid, Dulbecco's modified Eagle's Medium (DMEM), fetal bovine serum (FBS), sodium hydroxide (NaOH), Trypsin‐EDTA, penicillin‐streptomycin, all‐trans‐retinoic acid (ATRA), paraformaldehyde (PFA) and Triton X‐100 were purchased from Sigma‐Aldrich (UK). Dichloromethane (CH_2_Cl_2_), Dulbecco's phosphate‐buffered saline (PBS), DMEM/ F‐12, B‐27 Plus Neuronal Culture System GlutaMAX supplement, AlamarBlue Cell Viability Reagent, LIVE/DEAD Viability/Cytotoxicity Assay Kit, Alexa Fluor 488 Phalloidin and DAPI (4′ 6‐diamidino‐2‐phenylindole) were purchased from Thermo Fisher Scientific (UK). SH‐SY5Y (human neuroblastoma cell line CRL‐2266) was purchased from ATCC (USA).

### 2.2 Synthesis of Gelatin Methacryloyl

GelMA was synthesized using a previously reported protocol.^[^ ^29^ ^]^ Briefly, GelMA was prepared by dissolving 10 w/v% of gelatin powder in pre‐warmed PBS on a magnetic stirrer at 50 °C. After the gelatin was fully dissolved (stirred ≈1 h), 0.6 g of MA solution was added dropwise per 1 g of gelatin and stirred for 3 h. The solution was centrifuged at 3500 × g for 3 min to remove unreacted MA, and the supernatant was collected immediately. The solution was diluted with two volumes of 40 °C pre‐warmed PBS, and dialyzed at 40 °C against deionized water for 7 days using a 12 kDa MWCO dialysis membrane (Spectra/Por, Fisher Scientific). 5 M NaOH was used to adjust the pH to 7.4 and a 0.22 µm syringe filter (PES membrane, Starlab, UK) was used to filter the GelMA solution. The solution was kept at ‐80 °C overnight, lyophilised, and stored at ‐20 °C away from light and moisture.

### 2.3 Hydrogel Fabrication

GelMA pre‐polymer solution was prepared by dissolving 10 w/v% of GelMA in PBS at 40 °C. The GelMA/PEGDA solutions were prepared by adding 5 w/v% or 15 w/v% PEGDA powder with different molecular weights to 10 w/v% GelMA solution (**Table** **1**). Photoinitiator concentrations of 0.1/1, 0.2/2, and 0.3/3 Ru/SPS (mm/mm) were used. The photoinitiators were added and stirred immediately prior to photocrosslinking to avoid rapid redox reaction and precipitation.

**Table 1:** The composition of the hydrogels.

Sample	PEGDA Mn	GelMA% [w/v]	PEGDA% [w/v]
10G	/	10	/
10G+5P700	700	10	5
10G+15P700	700	10	15
10G+5P2000	2000	10	5
10G+15P2000	2000	10	15
10G+5P4000	4000	10	5
10G+15P4000	4000	10	15

Photocrosslinking was performed by a visible light system (UV‐LED Light Source, LC‐L1V5, HAMAMATSU, Japan) using a 405 nm wavelength, a light source‐sample distance of 10 cm, and an irradiation time of 5 min. Cylindrical hydrogel samples with a height of 5 mm and a diameter of 8 mm were fabricated and used in the SEM, mechanical, gel fraction, water content, swelling, and degradation characterization. 0.2/2 Ru/SPS (mm/mm) was used throughout except for mechanical studies.

### 2.4 NMR Spectra of GelMA

^1^H NMR spectroscopy (400 MHz, Bruker, Germany) was used to determine the methacrylate degree of the free amine group in the GelMA sample according to a previous study.^[^ ^30^ ^]^ Briefly, 20 mg of GelMA was dissolved in 1 mL D_2_O containing 0.05 wt.% 3‐(trimethylsilyl)propionic‐2,2,3,3‐d_4_ acid. The spectra were normalized to the phenylalanine signal (6.00–7.50 ppm), representing the concentration of gelatin. The lysine methylene signals (2.80–2.95 ppm) of gelatin spectra and GelMA spectra were used to calculate the degree of functionalization (DoF) using the following:

(1) $$DoF_{GelMA}\;\left(\%\right)=\left[1-\frac{Area\left(lysine\;in\;GelMA\right)}{Area\left(lysine\;in\;gelatin\right)}\right]\times 100\%$$

### 2.5 Morphological Characterisation

The morphology and pore structure of GelMA and GelMA/PEGDA hybrid hydrogels were observed by scanning electron microscopy (SEM, Hitachi S‐3000N, Japan). The samples were frozen at −80 °C for two days and lyophilised by a vacuum freeze dryer (−53 °C) overnight. The specimen surface was then coated (Q150R S, Quorum Technologies, UK) with gold/palladium (80:20) to obtain ≈a 10 nm thick coating. The images were analyzed using ImageJ software (National Institute of Health, USA).

### 2.6 Mechanical Characterisation

The mechanical property of the hydrogels was evaluated through compression testing (Instron 3344, Instron Corp, Wilmington, USA) using a 10 N load cell. Casted cylindrical hydrogel samples were soaked in PBS at 37 °C for 24 h to ensure swelling. Samples with different photoinitiator concentrations of 0.1/1, 0.2/2, and 0.3/3 Ru/SPS (mm/mm) were prepared. A compressive strain rate of 0.5 mm min^−1^ was used from 0% strain till the sample began to break. Four samples (n = 4) of each group were analyzed and the experiments were performed in duplicate.

### 2.7 Gel Fraction, Water Content, Swelling, and Degradation

The gel fraction of hydrogels was determined by measuring the changes in their dry weights after incubation. Samples (n = 5) were lyophilised for two days, and the initial dry weight was recorded (W_i_). The samples were incubated in PBS at 37 °C for 24 h and lyophilised to determine the final dry weight (W_g_). The gel fraction was calculated as:

(2) $$Gel\;fraction\;\left(\%\right)=\frac{W_{g}}{W_{i}}\times 100\%$$

The water content (WC) of hydrogels was determined by measuring the changes in their weights upon water absorption. Samples (n = 5) were incubated at 37 °C for 24 h in PBS, wiped with tissue paper to remove the excess water, and weighed (W_w_). The WC was calculated according to the following equation:

(3) $$WC\left(\%\right)=\frac{W_{w}-W_{i}}{W_{w}}\times 100\%$$

The swelling of the GelMA/PEGDA hybrid hydrogels was investigated by the gravimetric method. The lyophilised sample (n = 5) was weighed and immersed in PBS and incubated at 37 °C for a swelling time ranging from 0.5–24 h. At designated time points, samples were taken out of PBS to measure the weight (W_s_). Before weighing, the hydrogel was placed between two tissue papers to gently remove excess liquid. The swelling ratio (Q) was calculated as the following equation:

(4) $$Q=\frac{W_{s}}{W_{i}}$$

The degradation of the samples was assessed by weighing the initial lyophilised samples (W_i_; n = 4 for each time point) and immersing them in DMEM and incubating them at 37 °C for up to 3 weeks. The DMEM was replaced every two days. To determine the degradation kinetics, the swollen hydrogel samples were frozen at −80 °C for two days and then lyophilised at each time point. The final dry weight (W_d_) after incubation was measured. The extent of degradation was determined gravimetrically as the remaining weight according to the following:

(5) $$Remaining\;weight\left(\%\right)=\frac{W_{d}}{W_{i}}\times 100$$

### 2.8 Rheology Characterisation

The bioink rheological behavior was analyzed using an AR‐G2 rheometer (TA Instruments, USA) with a 20 mm diameter parallel plate, a 500 µm gap size, and a solvent trap. A flow sweep (viscosity vs shear rate) was performed from 0.1 to 1000 s^−1^ at 25 °C. The amplitude sweep (shear modulus vs strain) was between 0.01 and 1000% strain at 1 Hz and 25 °C to access the linear viscoelastic region (LVER). The temperature sweep (shear modulus vs temperature and viscosity vs temperature) was performed at a ramp rate of 1 °C min^−1^ at 1 Hz frequency, 1% strain, between 10–50 °C. The recoverability of the pre‐polymer solutions was checked by time sweeps (shear modulus vs time), which were performed at 1 Hz and 25 °C by using 1% and 1000% strain with 60 s equilibration time for each step. Frequency sweeps (shear modulus vs angular frequency) were performed between 0.1 and 100 rad s^−1^ with 10% strain at 25 °C to determine the storage (G′) and loss (G′′) modulus.

### 2.9 Printability

The printability of the bioink was performed using an extrusion‐based bioprinter (BIOX6, CELLINK, Sweden). A pneumatic temperature‐controlled printhead (25–27 °C) was used to deposit the bioinks onto a temperature‐controlled printing platform (25 °C). The bioink temperature was equilibrated at 25–27 °C for ≈30 min prior to printing. All bioinks were printed using a 25G tapered nozzle (250 µm inner diameter). The printing parameters of each group of bioinks were obtained through a printability study to ensure continuous and stable extrusion. Multi‐layer porous structures (n = 4) with dimensions of 10 mm × 10 mm × 1 mm, 0/90° lay‐down pattern, 4 layers, and 9% rectilinear infill density were fabricated for analysis. The structures were exposed to visible light photocrosslinking (405 nm, 5 min, 10 cm distance). The structures were imaged by light microscopy (VHX‐5000, Keyence, Japan), and their filament width, pore area, and printability parameter (Pr) were measured by ImageJ. The circularity and Pr was calculated according to the following:

(6) $$Circularity=4\pi \times \frac{Area}{Perimter^{2}}$$

(7) $$Pr=\frac{\pi /4}{Circularity}$$

### 2.10 Cell Culture and Bioprinting

SH‐SY5Y neuroblastoma cells (passage 4, ATCC CRL‐2266) were used and cultured with DMEM/F‐12 (1:1 v/v) cell culture medium supplemented with 10% fetal bovine serum (FBS), 1% penicillin/streptomycin, and 1% glutamine in an incubator (37 °C, 5% CO_2_, and 95% humidity). Media was refreshed every 3 days and cells were subcultured after reaching 80–90% confluency. Adherent cells were washed with PBS and detached using 0.25% trypsin‐EDTA solution.

The cell suspension was gently mixed with the pre‐polymer solutions and photoinitiators to obtain a 1 × 10^6^ cells mL^−1^ bioink. Cell‐laden constructs were bioprinted (10 mm × 10 mm × 1 mm, 4 layers, and 9% rectilinear infill density) using optimized parameters (Table S1, Supporting Information), followed by visible light photocrosslinking (405 nm, 5 min, 10 cm distance). The bioprinted constructs were incubated in a cell culture medium which was changed every 3 days.

### 2.11 Cell Viability, Metabolic Activity, and Morphology

Cell viability of bioprinted cell‐laden samples was observed using a live/dead viability assay kit on day 0 (immediately post‐bioprinting) and day 7. Following the manufacturer's instructions, the live/dead solution was prepared in PBS with a 1/2000 and 1/500 dilution of calcein‐AM and ethidium homodimer‐1, respectively. The samples (n = 3) were immersed in the live/dead solution and incubated for 25 min. The cells were imaged by confocal microscopy (SP8, Leica Microsystems, Germany), and the live and dead cells were counted using ImageJ. The ratio of live cells to the total cell number was used to determine cell viability, using a minimum of three images per sample.

The Alamar Blue assay assesses cellular metabolic activity and can be used as an indicator of cell proliferation. The samples (n = 4) were evaluated on days 1, 3, 5, 7, and 9. A 10% (v/v) Alamar Blue solution was added to the cell culture medium and incubated for 4 h. 150 µL of the sample solution was transferred to a 96‐well plate, and the fluorescence was measured (ex 530 nm/em 590 nm) using a plate reader (Synergy HT, BioTec, USA).

After 3 and 14 days of culture, cells were fixed and stained for confocal microscopy imaging (SP8, Leica Microsystems, Germany). The samples were rinsed twice with pre‐warmed PBS (37 °C), fixed in 10% PFA for 30 min, and permeabilized with 0.2% Triton X‐100 for 1 h at room temperature. The samples were blocked in a 10% (v/v) FBS solution and stained with 1:400 Alexa Fluor 488 phalloidin solution containing 1% FBS overnight at 4 °C, followed by 1:1000 DAPI staining for 2 h at room temperature in the dark. The samples were washed with PBS after each step. The stained samples were stored at 4 °C prior to imaging.

### 2.12 Statistical Analysis

Origin software (OriginLab, USA) was used for statistical analysis by performing a one‐way analysis of variance (ANOVA) with the Tukey test. Differences were considered statistically significant at ^*^ *p* < 0.05, ^**^ *p* < 0.01, and ^***^ *p* < 0.001. The data is presented as the mean ± standard deviation.

## 3 Results and Discussion

### 3.1 Methacrylation of Gelatin

**Figure 1:**
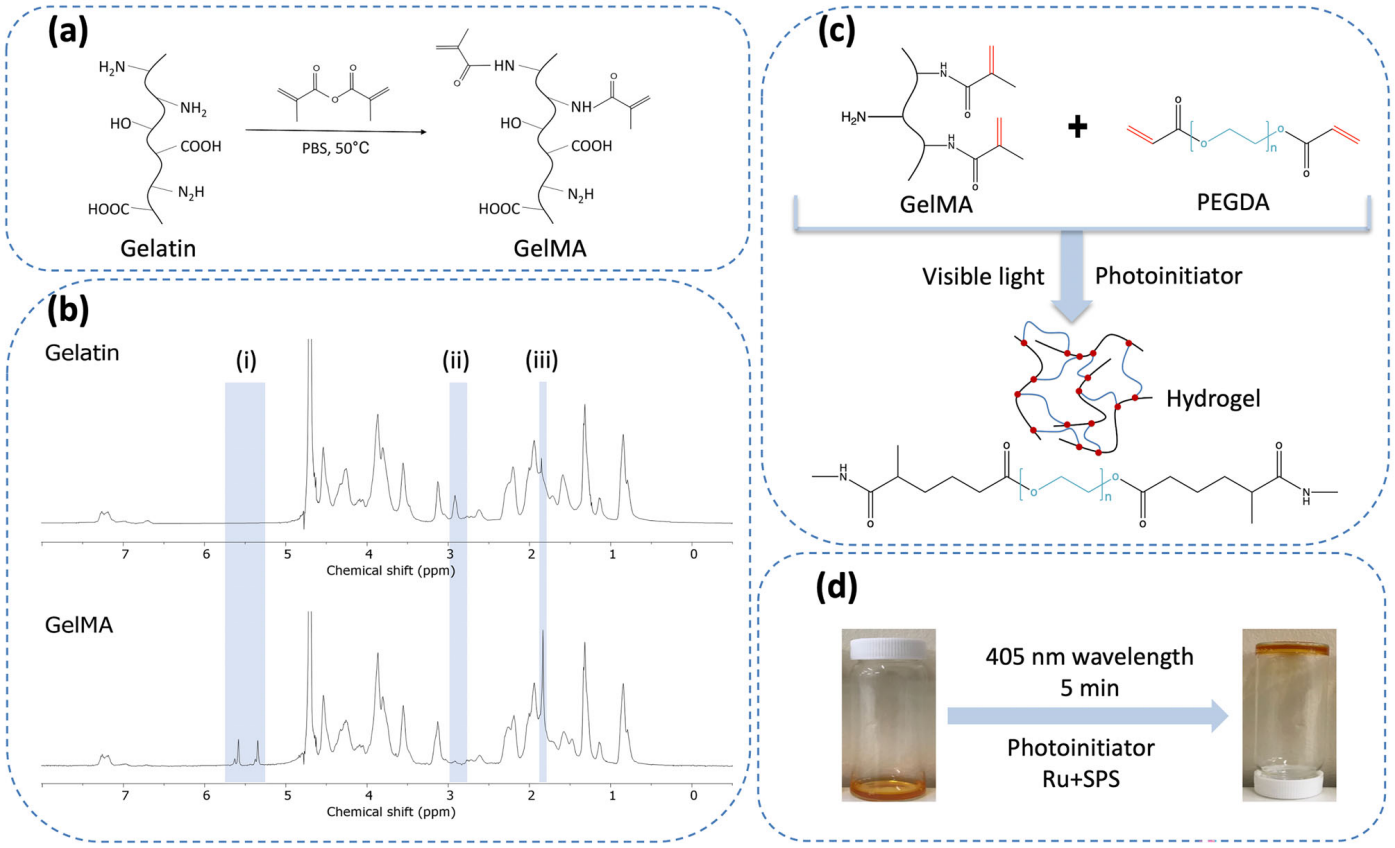
Hybrid GelMA/PEGDA hydrogel fabrication. a) Synthesis of GelMA. b) ^1^H NMR spectra of the GelMA (i) acrylic protons of methacryloyl grafts and hydroxyl lysine groups, (ii) methylene protons of unreacted lysine, and (iii) methyl protons of methacryloyl grafts. c) Schematic of the crosslinking mechanism of the GelMA/PEGDA hydrogels forming an interconnected covalently crosslinked hybrid network. d) GelMA/PEGDA hydrogel after photocrosslinking.

The polypeptide backbone of gelatin has multiple functional groups that can serve as grafting sites for other molecules, including amino (‐NH2), hydroxyl (‐OH), and carboxyl (‐COOH). The reaction mechanism is displayed in **Figure** **1**a. Briefly, methacrylic anhydride reacted with reactive amine and hydroxyl groups of amino acid residues to introduce an unsaturated bond on the gelatin molecular chain. Thus, GelMA can be crosslinked via free radical photopolymerisation in an aqueous solution with a photoinitiator. The degree of methacrylation can be controlled by the amount of methacrylic anhydride.^[^ ^30^, ^31^ ^]^

^1^H NMR spectrum was used to confirm the successful modification of MA into gelatin molecules. Gelatin and GelMA display complex spectra due to the presence of multiple amino acids in the peptide (Figure 1b). New signals appear at δ = 5.4 ppm and δ = 5.6 ppm in the GelMA spectrum, which are the peaks of the acrylic acid protons of the methacrylic acid functional group; the peak at 1.87 ppm corresponds to the methyl group of methacrylic acid. The decrease in the peak at δ = 2.9 ppm in the GelMA spectrum represents the reduction of lysine groups compared to gelatin. In addition, GelMA and gelatin exhibit similar chemical shifts and peak intensities, indicating that GelMA retains the structure and characteristics of gelatin. The DoF determines the maximum crosslinking density, thereby affecting the stiffness of the hydrogel.^[^ ^31^ ^]^ In this study, the DoF was calculated to be 72.26%, which is in the high DoF range. The GelMA/PEGDA pre‐polymer solution was photopolymerised with Ru/SPS and 405 nm visible light causing hydrogel formation (Figure 1c,d). Under light exposure, the photoinitiator suffers a cleavage mechanism producing free radicals that react with the acryloyl groups producing a covalently bonded crosslinked polymer.^[^ ^32^ ^]^

### 3.2 Hydrogel Characterisation

**Figure 2:**
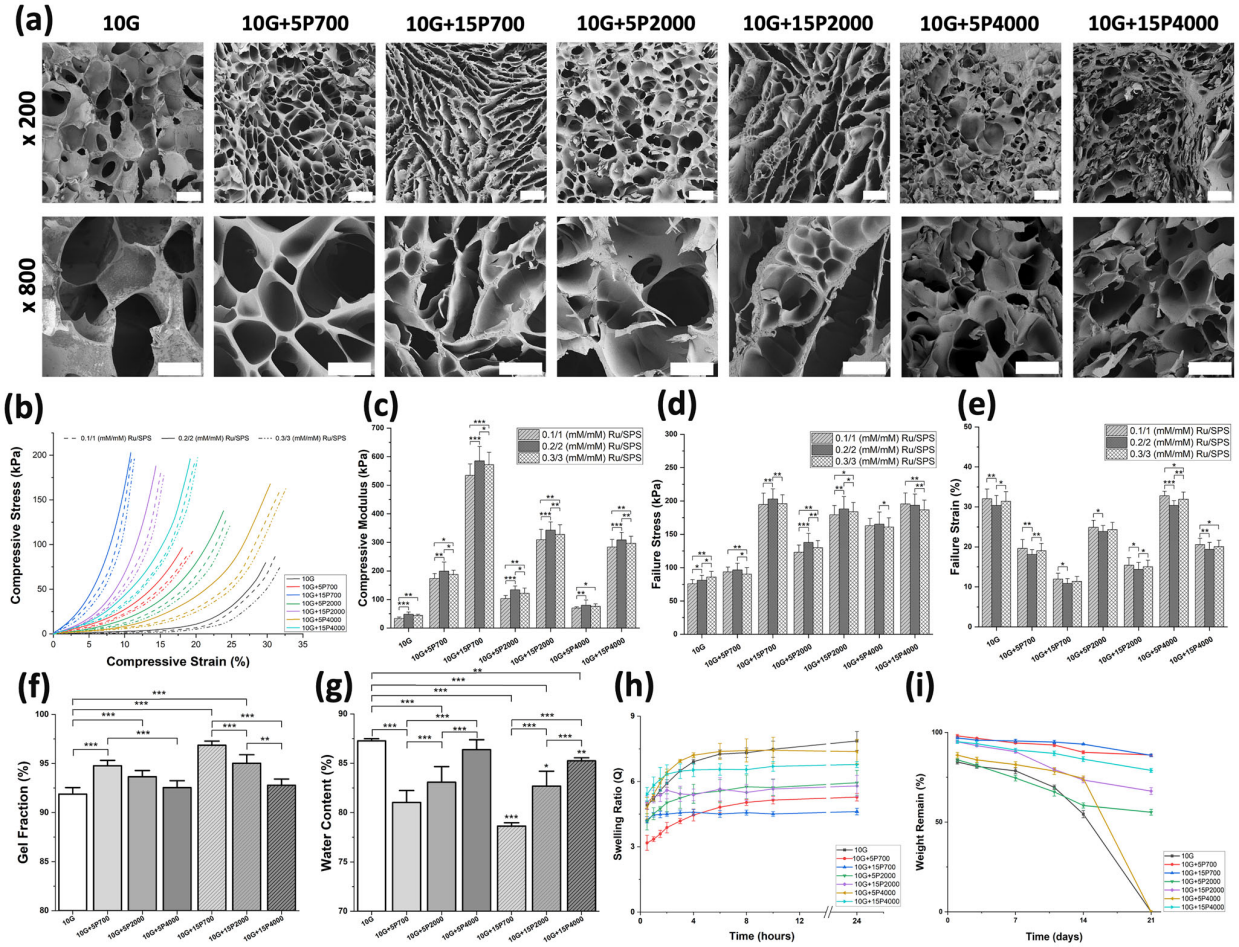
Physical characteristics of the hydrogels. a) SEM images of the internal microstructure of hybrid hydrogels (x200: scale = 200 µm; x800 scale = 100 µm). b) Stress–strain curves, c) mechanical compression modulus, d) failure stress, and e) failure strain. f) Gel fraction, g) water content in hydrogels after 24 h, h) swelling ratio, and i) weight remain after degradation.

A 10 w/v% GelMA was utilized due to its extrusion‐based printability,^[^ ^33^, ^34^ ^]^ however, due to the poor mechanical properties and inappropriate degradation rate for PNI applications, specific concentrations of PEGDA (5 and 15 w/v%) with different molecular weights (Mn = 700, 2000, 4000) were added to identify potentially suitable bioinks. SEM images of the internal structure of the hydrogels are presented in **Figure** **2**a. The porosity of the hydrogels was semi‐quantitatively assessed based on SEM images (Figure S1 and Table S2, Supporting Information). The GelMA hydrogel has a dense porous structure and the addition of PEGDA increases the porosity. Higher concentrations of PEGDA result in a less porous structure. Furthermore, as the PEGDA molecular weight increases, a slight trend of higher porosity is observed. Although the PEGDA 4000 hydrogels exhibit a more irregular pore structure. By changing the PEGDA concentration and molecular weight, the hydrogel network formation can be modulated. Similar results have been observed in the literature.^[^ ^23^, ^24^, ^28^ ^]^ However, the freeze‐drying processing of the hydrogels for SEM imaging can introduce artifacts that mask the actual microstructure.^[^ ^15^ ^]^ For example, the PEGDA 4000 samples show a smaller and more solid structure after freeze‐drying compared to the other groups suggesting that the original microstructure was not intact. Thus, further assessment is required to understand the microstructure of the hybrid hydrogels.

The effect of photoinitiator concentration on the formation of the hydrogels was assessed by uniaxial static compression tests to select the optimal parameters for hydrogel preparation (Figure 2b–e). The mechanical compression stress‐strain curves are shown in Figure 2b. The compressive modulus of the hydrogels with a photoinitiator concentration of 0.2/2 (mm/mm) Ru/SPS is slightly higher than the hydrogels with 0.1/1 and 0.3/3 (mm/mm) Ru/SPS photoinitiator concentrations. The results demonstrated that 0.2/2 (mm/mm) Ru/SPS was able to sufficiently crosslink the macromolecular monomers of GelMA and PEGDA, thus being used for all experimental studies. Lim et al.^[^ ^35^ ^]^ confirmed that GelMA hydrogels prepared with a photoinitiator concentration of 0.2/2 or 0.3/3 (mm/mm) Ru/SPS had similar polymerization rate, compression modulus, and sol fraction indicating full crosslinking of the GelMA macromonomers.

Furthermore, the compressive Young's modulus of the GelMA/PEGDA group is significantly higher than that of the pure GelMA group (Figure 2c). This can be attributed to the high degree of crosslinking interactions between the GelMA and PEGDA networks. The elastic modulus of hydrogels ranges from ≈50 kPa (10G) to 600 kPa (10G+15P700 ), making them suitable for a variety of tissues, including nerves, skin, and articular cartilage.^[^ ^36^ ^]^ The addition of PEGDA increases the compressive stress at failure compared to the pure GelMA group (Figure 2d,e). The molecular weight of the PEGDA has a significant effect on the mechanical properties. The higher PEGDA molecular weight can lead to a decrease in elastic modulus, higher failure stress, and higher failure strain of the hybrid hydrogel. Increasing the concentration of PEGDA from 5 to 15 w/v% at all molecular weights made the hybrid hydrogel stiffer with higher failure stress and a decrease in failure strain. Sala et al.^[^ ^22^ ^]^ showed that PEGDA700 hydrogels have a higher compressive modulus than PEGDA3400 at the same concentration due to more short chains contributing to a higher crosslinking density. As molecular weight increases, crosslinking density decreases due to fewer and longer chains. Higher polymer concentration increases hydrogel stiffness but also brittleness. Thus, the hybrid hydrogel's mechanical properties increase as PEGDA molecular weight decreases and concentration increases.

The gel fraction is the percentage of pre‐polymer converted into a polymer network, indicating the efficacy of the crosslinking process. All hydrogels present high gel fractions ranging from 92% to 97%, and the addition of PEGDA increases the gel fraction compared to the pure GelMA hydrogel (Figure 2f). The hybrid hydrogel with higher PEGDA concentration and lower molecular weight has a higher gel fraction, indicating that the 10G+15P700 group forms the highest chemically crosslinked network, and the 10G+5P4000 group has the lowest crosslinked network.

The water content of the hydrogels was determined by measuring the change in weight after water absorption. As shown in Figure 2g, the addition of PEGDA reduced the water content compared to the pure GelMA hydrogel. The hybrid hydrogel with lower PEGDA concentration and higher molecular weight had significantly higher water content.

The swelling ratio of the hydrogels after photocrosslinking was determined as this can affect the physical and biological properties (e.g., ligand presentation, pore architecture, stiffness, mobility, and solute diffusion) (Figure 2h).^[^ ^37^, ^38^ ^]^ The swelling ratio of all hydrogels significantly increases in the first 4 h and then gradually plateau. However, GelMA exhibits an increase in swelling at 24 h. The addition of PEGDA decreases the swelling ratio. After the samples reach the swelling equilibrium, the hybrid hydrogels with lower PEGDA concentration and higher molecular weight have a higher swelling ratio, with 10G+5P4000 being similar to GelMA. This is attributed to the decreased crosslinking density with a decrease in PEGDA concentration. Similarly, increasing the molecular weight of PEGDA reduces the molar percentage of PEGDA, thus, reducing the crosslinking density of the hydrogel and enhancing its swelling capacity. These results are consistent with the water content results of the hydrogels (Figure 2g).

Degradation curves were obtained by measuring the mass loss of GelMA and GelMA/PEGDA hydrogels over time, as shown in Figure 2i. The GelMA/PEGDA700 hydrogel showed the slowest degradation rate compared to the other hydrogels. Pure GelMA and 10G+5P4000 hybrid hydrogels were completely degraded after 2 weeks; however, hydrogels from the other groups retained their structural integrity shape, and their weight remained over 50% for up to 3 weeks. The degradation rate of the GelMA only hydrogel agrees with a study by Zhao et al.^[^ ^39^ ^]^ The addition of PEGDA delayed the in vitro degradation of the hybrid hydrogels, thus overcoming a disadvantage of pure GelMA hydrogels.

The mechanical properties, swelling, and degradation rate of the hydrogel should match the regeneration rate of the peripheral nerve tissue to ensure the integrity of the NGCs. The incorporation of different concentrations and molecular weights of PEGDA allows the tuning of hydrogel properties. In summary, increasing the PEGDA concentration led to a higher crosslinking density resulting in a dense and lower porosity network, thus increasing the elastic modulus, failure stress, and gel fraction but decreasing the water content, swelling ratio, and degradation rate. Alternatively, increasing the PEGDA molecular weight from 700 to 4000 leads to fewer short chains and longer chains, resulting in a lower crosslinking density. Subsequently, a decrease in elastic modulus and gel fraction was observed but the failure stress and strain, water content, swelling ratio, and degradation rate all increased.

### 3.3 Bioink Rheology

**Figure 3:**
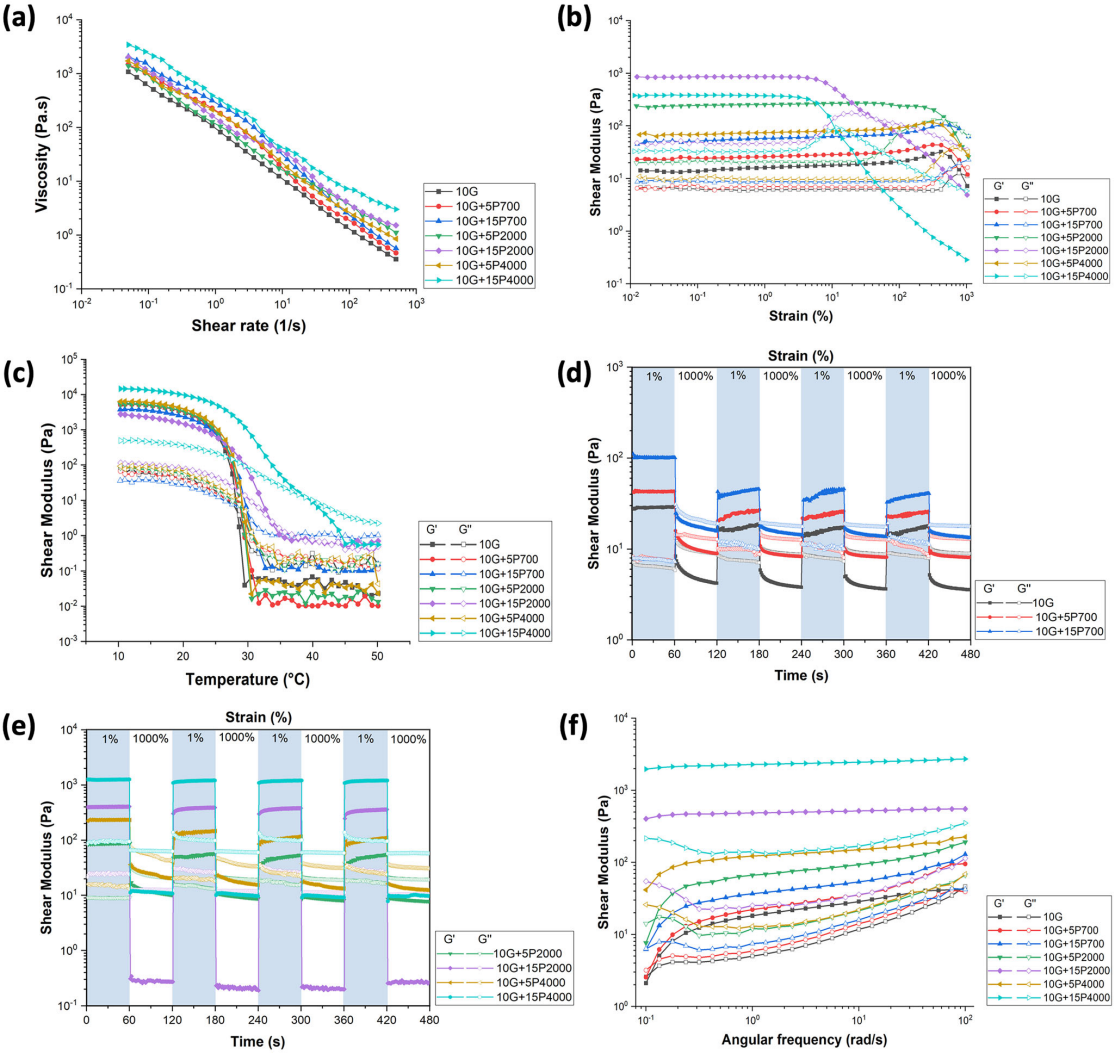
Rheology of the GelMA/PEGDA hybrid bioinks. a) Shear rate sweep showing shear‐thinning behavior. b) Strain sweep results show LVER and gel to fluid‐like transition. c) Temperature sweep ranging from 10–50 °C. d,e) Time sweeps with alternative strain between low stain (1%) and high strain (1000%) presenting the transition ability from liquid‐like flow to solid‐like elastic shape retention. f) Frequency sweep showing the correlation between angular frequency and modulus under 25 °C.

Typically, bioink rheology for extrusion‐based bioprinting should allow flow initiation, shear thinning, and self‐recovery to enable self‐supporting and high fidelity structures.^[^ ^11^ ^]^ Thus, rheology can be used as a tool to determine printability and and optimize printing parameters. Shear thinning, a non‐Newtonian behavior, occurs when viscosity decreases as the shear rate increases. This happens due to polymer chain disentanglement and arrangement of chains along the flow direction, reducing internal friction and viscosity. The removal of the force allows chain re‐entanglement an increase in viscosity and a shape stable bioink.^[^ ^11^, ^40^ ^]^ **Figure** **3**a shows that all the bioinks exhibit shear thinning behavior, indicating that the pre‐polymer solutions exhibit fluid‐like behavior at high shear and hence are suitable for extrusion. The addition of PEGDA increases the viscosity of the bioinks in a concentration and molecular weight dependent manner. Higher concentrations and molecular weight reduce the free volume for polymer chain mobility and increase chain entanglement, resulting in higher viscosity.

All the bioinks exhibit viscoelastic behavior in the amplitude sweep (Figure 3b). The linear viscoelastic region (LVER) represents the lowest strain range where testing can be conducted without compromising the sample structure. The storage modulus, G', representing the elastic behavior, and the loss modulus, G'', constituting the viscous component can be used to characterize the viscoelastic properties. The LVER was observed in the low strain region (10^−2^‐10^0^%) in all hydrogels. The G' is greater than G'' in the LVER, indicating that the bioink is a viscoelastic solid material. As the strain increases, G'' becomes higher than G', the viscous component dominates, and a fluid behavior is exhibited. Higher concentrations and molecular weight of PEGDA increased the G' value in the LVER.

The G' and G'' of the bioink in a temperature sweep between 10 and 50 °C are shown in Figure 3c. As the temperature increases, both G' and G'' decrease for all samples. The increasing temperature can efficiently break intermolecular interactions in GelMA which is in a physically gelled state at low temperatures. GelMA exhibits a similar gel‐sol transition temperature (≈29 °C) to the low‐concentration hybrid bioinks. However, as the PEGDA concentration increased, the gel‐sol transition shifted to higher temperatures in a molecular weight dependent manner (10G+15P700 ≈32 °C; 10G+15P2000 ≈35 °C; 10G+15P4000 ≈39 °C). Furthermore, the G' and G'' of the G+15P4000 bioink are higher than the other bioinks due to the high concentration and molecular weight. The results can be attributed to the addition of PEGDA increases the molecular distance and decreases the interaction between molecules, thus, decreasing the relaxation time of molecular chains.^[^ ^22^ ^]^ Figure S2 (Supporting Information) shows the viscosity decreases as the temperature increases.

During extrusion, the bioink goes from a high to low strain regime as the bioink flows through the nozzle onto the platform. The bioink should rapidly transform from a fluid‐like to a solid‐like state in response to the change in strain to allow shape recovery. The time dependence recovery of G' and G'' at low strain (1%) and high strain (1000%) shows that G' is greater than G'' for all samples at low strain (Figure 3d,e). The G' decreases immediately below the G'' value at high strain, indicating a rapid transition from a solid‐like to a liquid‐like phase. The bioink responds quickly to changes in applied shear strain and recovers rapidly following the removal, demonstrating self‐recovery characteristics. Among them, 10G+15P2000 and 10G+15P4000 showed outstanding reversibility by not observing significant changes in four cycles. Higher G'' may negatively impact the flow of bioink during extrusion at high strain. A higher G' can better maintain the shape fidelity of the filament on the platform. The addition of PEGDA enhanced the G' and G'' values. Higher PEGDA concentration and larger molecular weight resulted in increased G' and G'' values.

Oscillatory rheology in the LVER was used to investigate the mechanical strength of the bioinks. Figure 3f indicates that all bioinks exhibit frequency‐dependent behavior with both the G' and G'' increasing with frequency. Compared to G'', G' remains relatively constant over the frequency range tested, however, as the frequency increases, G' and G'' tend to intersect. The higher concentrations of PEGDA and larger molecular weight result in a higher G' and G'' in the hybrid bioinks.

### 3.4 Printability

**Figure 4:**
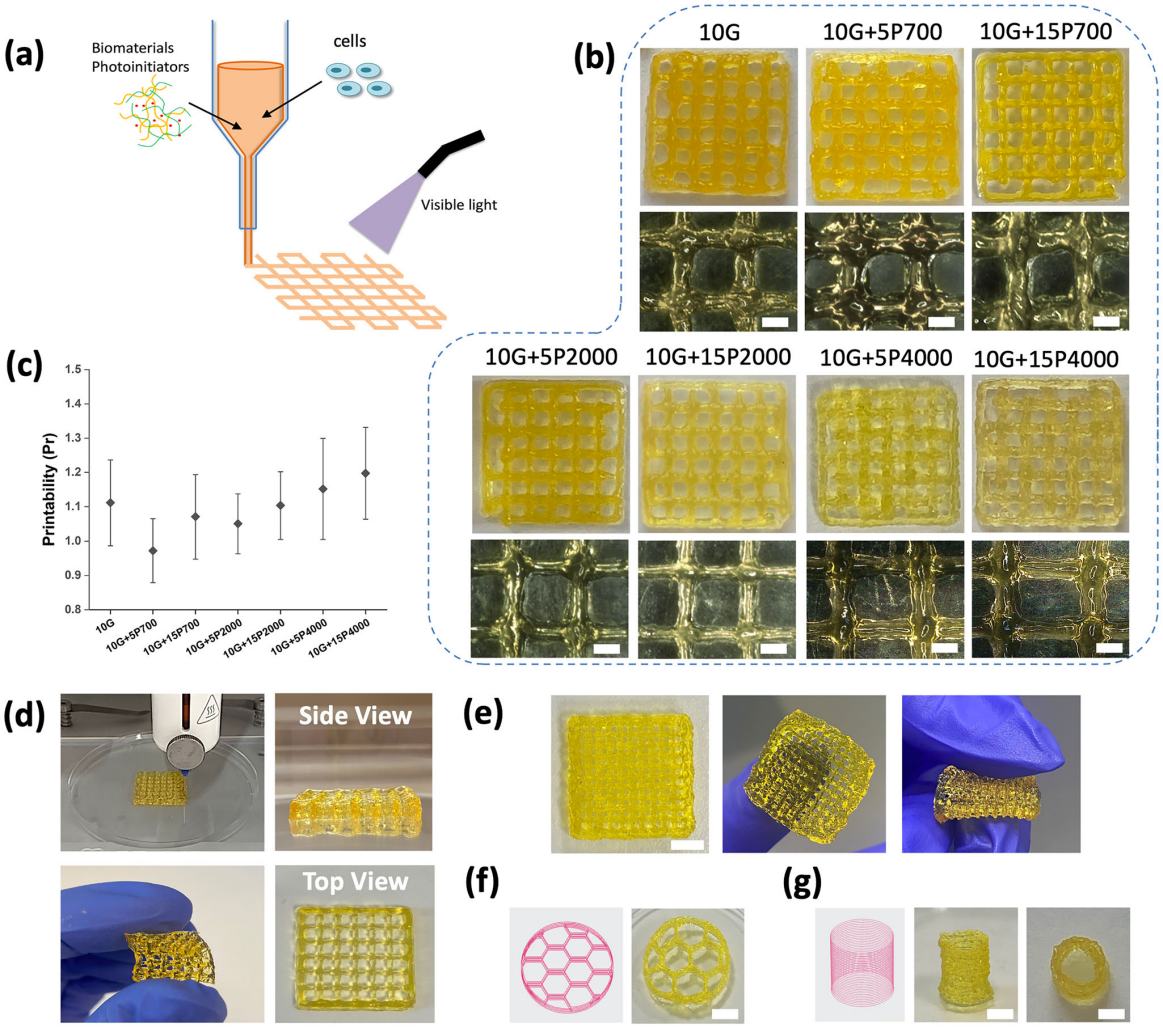
Printability assessment. a) Schematic of bioprinting and photocrosslinking process. b) Printed constructs and corresponding representative microscope images (scale = 1000 µm) and c) printability analysis. Examples of printed structures with good shape fidelity using 10G+5P700, d) images of the printing process with side and top‐view of multi‐layer structure (10 × 10 mm, 4 layers, 9% infill density) and structure being compressed, e) high‐density lattice showing intact folding (20 × 20 mm, 6 layers, 25% infill density) (scale = 5 mm), f) honeycomb structure (10 mm diameter, 2 mm height, 9% infill density) (scale = 3 mm), and g) hollow cylinder (6 mm diameter, 8 mm height, 2% infill density, 40 layers) (scale = 3 mm).

The rheology of the bioinks shows shear‐thinning and self‐recovery behaviour indicating potential suitability for 3D bioprinting applications. The printability of the hybrid GelMA/PEGDA bioinks was evaluated through morphological observation after visible light photocrosslinking (**Figure** **4**). To ensure the quality and shape fidelity of the filaments, several parameters (nozzle diameter, pressure, print speed, temperature) were adjusted to determine the optimal processing parameters (Table S1, Supporting Information). As the PEGDA concentration and molecular weight increased, higher printing pressure, slower printing speed, and a slight increase in temperature were generally required for suitable extrusion. Using the printing parameters all bioinks could be extruded with stable and uniform filaments and were successfully printed into stable multi‐layer 3D structures (Figure 4b).

A semi‐quantitative assessment based on the ideal square or rectangular shaped pore the in the x‐y plane of a 0/90° lay‐down pattern (Pr = 1) was used to determine printability (Figure 4c).^[^ ^11^, ^41^ ^]^ A Pr < 1 and Pr > 1 correspond to a rounded shape caused by insufficient gelation and irregular lateral geometry caused by excessive gelation, respectively. The ideal Pr = 1 results in smooth and uniform filament extrusion with suitable gelation. The greater the difference from this ideal value, the poorer the printability, shape fidelity, and irregularity of the filament structure. The 3D printed GelMA bioink showed good printability, Pr ≈1.1, at 25 °C. Increasing the printing temperature to 26 °C resulted in droplets forming at the nozzle tip due to the sol‐gel transition temperature, thus unprintable. Compared to the other bioinks, GelMA has the lowest printing pressure, as the more viscous GelMA/PEGDAs require higher pressure for the bioink to flow. The addition of PEGDA700 and PEGDA2000 improves the printability of pure GelMA, with a Pr∼1. This is because the rheological properties of the bioinks (e.g., viscosity, storage modulus, and shear recovery) are more suitable for extrusion bioprinting. However, the addition of PEGDA4000 (Pr∼1.2) reduces the printability with irregular horizontal geometries observed and requires significantly higher printing pressures (110–200kPa) and slower extrusion speeds (4–5 mm s^−1^) to fabricate stable structures due to the high viscosity. The higher printing pressure can also lead to increased shear stress, which may cause permanent damage to cells in the bioinks.^[^ ^41^ ^]^

These results indicate that the GelMA/PEGDA has good printability and supports the bioprinting of multi‐layer structures. The bioinks show high fidelity enabling the fabrication of high infill density, honeycomb, and hollow cylinder structures (Figure 4d–g). Furthermore, the printed and crosslinked structures could be handled, compressed, rolled, and returned to the original shape. The extrusion‐based bioprinting strategy in this study shows feasibility for fabricating tubular structures within the typically observed range of inner diameters, 1.5–30 mm, observed in commercially available NGCs.^[^ ^42^ ^]^

### 3.5 Bioprinting and Cytocompatibility of SH‐SY5Y Cells

The GelMA/PEGDA bioinks containing SH‐SY5Y cells were bioprinted and photocrosslinked using the optimized printing parameters to fabricate multi‐layer lattice‐based constructs. The cell viability, proliferation, and morphological properties of SH‐SY5Y cells were assessed to determine cytocompatibility and the attractiveness of the hybrid bioinks in peripheral nerve tissue engineering applications. Furthermore, the role of PEGDA concentration and molecular weight on cell behavior was elucidated.

**Figure 5:**
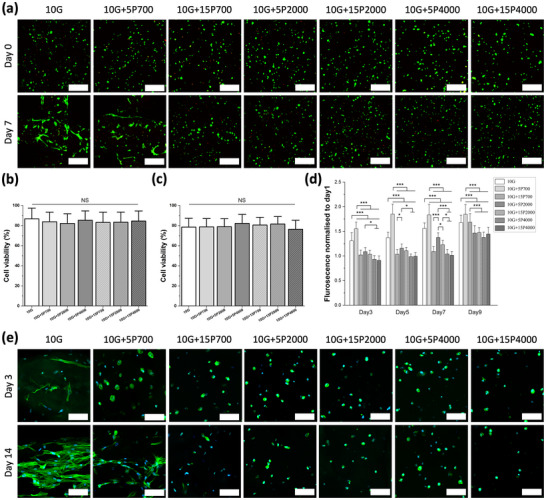
Cytocompatibility studies of bioprinted SH‐SY5Y cells. a) Representative confocal microscopy images showing live/dead results immediately after bioprinting and photocrosslinking (scale = 300 µm; green = live and red = dead). Cell viability b) immediately post‐bioprinting and c) after 7 days of cell culture. d) Cell proliferation results normalized to day 1. e) Confocal microscope images showing cell morphology in the bioprinted constructs at days 3 and 14 (scale = 100 µm; blue = nuclei and green = actin).

Cell viability was evaluated by live/dead assay immediately after bioprinting and photocrosslinking, on day 0, and on day 7 (**Figure** **5**a–c). SH‐SY5Y cells were uniformly distributed in the matrix, and all groups showed high cell viability (>80%) immediately post‐printing with no significant difference between groups. This suggests that the shear‐thinning behavior of the bioink is not harmful to short‐term cell viability. In addition, cell viability of all groups remained above 75% after 7 days of in vitro culture, indicating high cytocompatibility of the GelMA and GelMA/PEGDA hydrogels. These results agree with studies showing high cell viability in GelMA/PEGDA hydrogels.^[^ ^7^, ^24^, ^25^, ^43^ ^]^

The Alamar blue assay was used to assess the metabolic activity of the SH‐SY5Y cells, with the values normalized to day 1 (Figure 5d). All samples showed a significant increase in cell number within 9 days. Similar cell numbers were observed (Figure 5c), but a higher metabolic activity was observed in the pure GelMA and 10G+5P700 groups, especially on days 3, 5, and 7. However, the 10G+15P700 group had a significantly smaller fluorescence compared to the 10G+5P700 group. In addition, higher molecular weight PEGDA with the same GelMA/PEGDA ratio had a negative effect on cell proliferation. Although hydrogels containing PEGDA700 have higher stiffness than PEGDA2000 and PEGDA4000, the greater capacity for metabolic activity may be because the stiffness of the hydrogel affects different cell types in different ways. Interestingly, the pure GelMA group and the 10G+5P700 group showed sustained cell metabolic activity over 9 days, whereas in the other groups, cell metabolic activity did not increase substantially on day 7 but increased significantly on day 9. Figure 5a also shows that cells in the pure GelMA and 10G+5P700 group occupied a larger area of the hydrogel and began to spread at day 7, which also supports the quantitative cell proliferation results. The results showed that all groups of hydrogels were able to promote cell proliferation over time.

The assessment of cell morphology showed that on day 3, the cells began to spread in the pure GelMA group, while the cells in the other groups exhibited a rounded morphology (Figure 5e). By day 14 the cells in GelMA and 10G+5P700 groups showed an elongated, branched, and more spreading morphology. The SH‐SY5Y cells spread in the printing direction of the filaments and exhibited anisotropic characteristics. The only PEGDA containing bioink that allowed cells to spread was the 10G+5P700, whilst the cells in the higher concentration and molecular weight hydrogels remained rounded. No change in cell morphology was observed at day 21 (Figure S3, Supporting Information).

GelMA as a derivative of collagen contains the cell adhesion motif, arginine‐glycine‐aspartic acid (RGD), that promotes cell attachment and spreading of encapsulated cells.^[^ ^15^ ^]^ GelMA hydrogels can promote neurite extension and neuronal network formation (neuronal cell survival, migration, and differentiation) under appropriate culture conditions.^[^ ^7^, ^44^ ^]^ Akcay and Luttge^[^ ^45^ ^]^ observed that SH‐SY5Y cells were able to survive being embedded in GelMA, whereas the PEGDA only hydrogels didn't support SH‐SY5Y cell survival. The current study complements this conclusion by observing that SH‐SY5Y cells were able to survive in the GelMA/PEGDA hydrogels, but had lower metabolic activity compared to GelMA only, indicating that the presence of GelMA and cell binding motifs is crucial for cell activity.

The limited cell proliferation and spreading observed in the PEGDA containing hydrogels, except 10G+5P700, is potentially related to the dense and highly crosslinked hydrogel network. Although no major morphological differences were observed between PEGDA containing hydrogels. They display distinct physical properties. The 10G+5P700 hydrogel exhibited cell proliferation and spreading but increasing the PEGDA concentration negatively impacted the cell response by increasing network density and reducing the free internal volume. Additionally, the PEGDA700 hydrogels have the highest stiffness whilst GelMA is the lowest. However, cells were able to spread in the GelMA and 10G+5P700 hydrogels suggesting that hydrogel stiffness was not the dominant factor in affecting cell behaviour. Alternatively, the high swelling capacity of the higher molecular weight PEGDA hybrid hydrogels (10G+5P4000), similar to GelMA alone, may reduce the ligand density (e.g., RGD) available for cell attachment.^[^ ^37^ ^]^ Furthermore, the presence of the PEGDA network can obscure the presentation of these ligands for cells. Subsequently, the low swelling of 10G+5P700, although densely crosslinked, may more easily present ligands for cell attachment. The rounded morphology and low proliferation rate of the cells in the high concentration and molecular weight hybrid hydrogels indicate that the cells have entered a quiescent state with the cells exiting the cell cycle but are still able to re‐divide upon stimulation.^[^ ^46^ ^]^ This may be caused by the poor diffusion of nutrients and signal molecules due to the dense structure of the hybrid hydrogel, thus the cells are deficient in growth factors and nutrients.

The cell behavior in PEGDA hybrid hydrogels is inconclusive. Sala et al.^[^ ^22^ ^]^ indicated that the viability of NIH‐3T3 fibroblasts on PEGDA3400 was slightly higher than that of PEGDA700. They suggested that cells preferred hydrogels with a larger mesh and lower Young's modulus. Conversely, Son and Lee^[^ ^23^ ^]^ showed that the chondrocyte proliferation rate of PEGDA3400 was higher than that of PEGDA6000 and PEGDA20000. However, both studies utilized different biomaterials, cell lines, and cell seeding rather than encapsulation as in this study, thus, direct comparisons are difficult to elucidate due to the distinct cell behavior in 2D and 3D environments. Further investigation is required to understand the PEGDA concentration and molecular weight dependencies on cell response and to evaluate a bioink with lower total polymer concentration and higher cell densities. Additionally, as a preliminary study the bioinks were bioprinted in only a lattice structure, future studies will assess the role of hollow tubular structures.

## 4 Conclusion

This study evaluated the effect of PEGDA concentration and molecular weight on the development of a long‐term stable and hybrid visible light photocrosslinked GelMA/PEGDA bioink. The results showed that the molecular weight and concentration could be used to tune the physical and biological properties of the hydrogels. As the concentration of PEGDA increased, the mechanical properties of the hybrid hydrogels increased, whilst the swelling and degradation rates decreased. Conversely, as the molecular weight of PEGDA increased, the mechanical properties decreased, whilst the swelling and degradation rates increased. The 10G+5P4000 hydrogel had the most similar properties to pure GelMA. The inclusion of PEGDA altered the hydrogel network formation creating a more densely crosslinked structure. Low PEGDA concentration and molecular weight resulted in a more uniform pore network.

All the bioinks had shear thinning and self‐recovery properties suitable for extrusion‐based bioprinting and enabling the fabrication of high fidelity, manipulable, and stable cell‐laden constructs. The addition of PEGDA700 and PEGDA2000 increased printability whilst PEGDA4000 slightly decreased printability. The bioinks showed high cytocompatibility after bioprinting and photocrosslinking. SH‐SY5Y cells in the GelMA and 10G+5P700 hydrogels showed proliferation and spreading in the printing direction. However, this was restricted in high concentration and molecular weight PEGDA bioinks due to the dense hydrogel network resulting in a rounded cell morphology and reduced proliferation.

Tailoring the PEGDA molecular weight and concentration offers precise adjustment of hydrogel properties that can be tuned for specific cell types. However, further investigation is required to understand the relationship between GelMA/PEGDA concentration and molecular weight dependent properties and SH‐SY5Y cell behavior, especially with lower total polymer content, higher cell densities, and longer culture periods. In conclusion, this study demonstrates that GelMA/PEGDA bioinks with SH‐SY5Y are printable and cytocompatible with potential for neuronal tissue engineering applications.

## Conflict of Interest

The authors declare no conflict of interest.

## Author Contributions

H.Y. performed conceptualisation, methodology, investigation, validation, formal analysis, visualization, wrote, reviewed, and edited the final manuscript. Y.W. performed methodology, investigation, validation, wrote, reviewed, and edited the final manuscript. S.F. performed investigation, wrote, reviewed, and edited the final manuscript. C.V. performed conceptualisation, methodology, investigation, validation, supervision, project administration, wrote, reviewed, and edited the final manuscript. P.B. performed conceptualisation, methodology, validation, resources, supervision, project administration, funding acquisition, wrote, reviewed, and edited the final manuscript.

## Supporting information



Supporting Information

## Data Availability

The data that support the findings of this study are available from the corresponding author upon reasonable request.
