# Supplementary material for: Bioprinting of GelMA/PEGDA Hybrid Bioinks for SH‐SY5Y Cell Encapsulation: Role of Molecular Weight and Concentration
Source: Macromol Biosci. 2025 Mar 25;25(6):2400587. doi: 10.1002/mabi.202400587 (PMC12169505; doi:10.1002/mabi.202400587)
Supplement: Supplementary file 1 — Supporting Information [file MABI-25-2400587-s001.docx]

**Bioprinting of GelMA/PEGDA hybrid bioinks for SH-SY5Y cell encapsulation: role of molecular weight and concentration**

Hexin Yue^1*^, Yaxin Wang^1^, Samantha Fernandes^1^, Cian Vyas^2*^, Paulo Bartolo^2*^

^1^ Department of Mechanical, Aerospace and Civil Engineering, University of Manchester, Manchester, M13 9PL, United Kingdom

^2^ Singapore Centre for 3D Printing, School of Mechanical and Aerospace Engineering, Nanyang Technological University, 639798, Singapore

*Corresponding authors:

hexin.yue@manchester.ac.uk, cian.vyas@ntu.edu.sg, pbartolo@ntu.edu.sg

**Table S1.** The printing parameters for bioinks.

| **Sample** | **Temperature (°C)** | **Pressure (kpa)** | **Speed (mm/s)** |
| --- | --- | --- | --- |
| 10G | 25 | 40-90 | 5-9 |
| 10G+5P700 | 25 | 70-110 | 5-9 |
| 10G+15P700 | 25 | 90-130 | 5-9 |
| 10G+5P2000 | 25 | 80-110 | 5-7 |
| 10G+15P2000 | 26 | 90-130 | 5-7 |
| 10G+5P4000 | 26 | 110-170 | 4-5 |
| 10G+15P4000 | 27 | 130-200 | 4-5 |


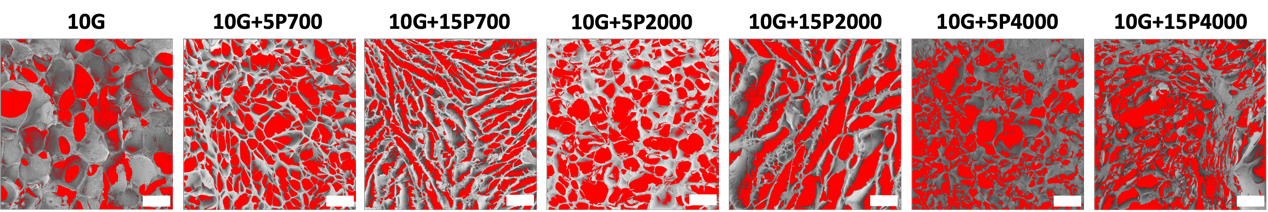
**Figure S1.** SEM images of hybrid hydrogels analysed by ImageJ thresholding to semi-quantitatively calculate porosity (scale = 200 µm).

**Table S2.** The porosity of the freeze-dried hydrogels.

| **Sample** | **Porosity (%)** |
| --- | --- |
| 10G | 25.135 ± 10.547 |
| 10G+5P700 | 41.845 ± 14.559 |
| 10G+15P700 | 37.046 ±10.819 |
| 10G+5P2000 | 47.121 ± 12.035 |
| 10G+15P2000 | 43.011 ± 10.587 |
| 10G+5P4000 | 39.667 ± 17.207 |
| 10G+15P4000 | 38.477 ± 14.123 |


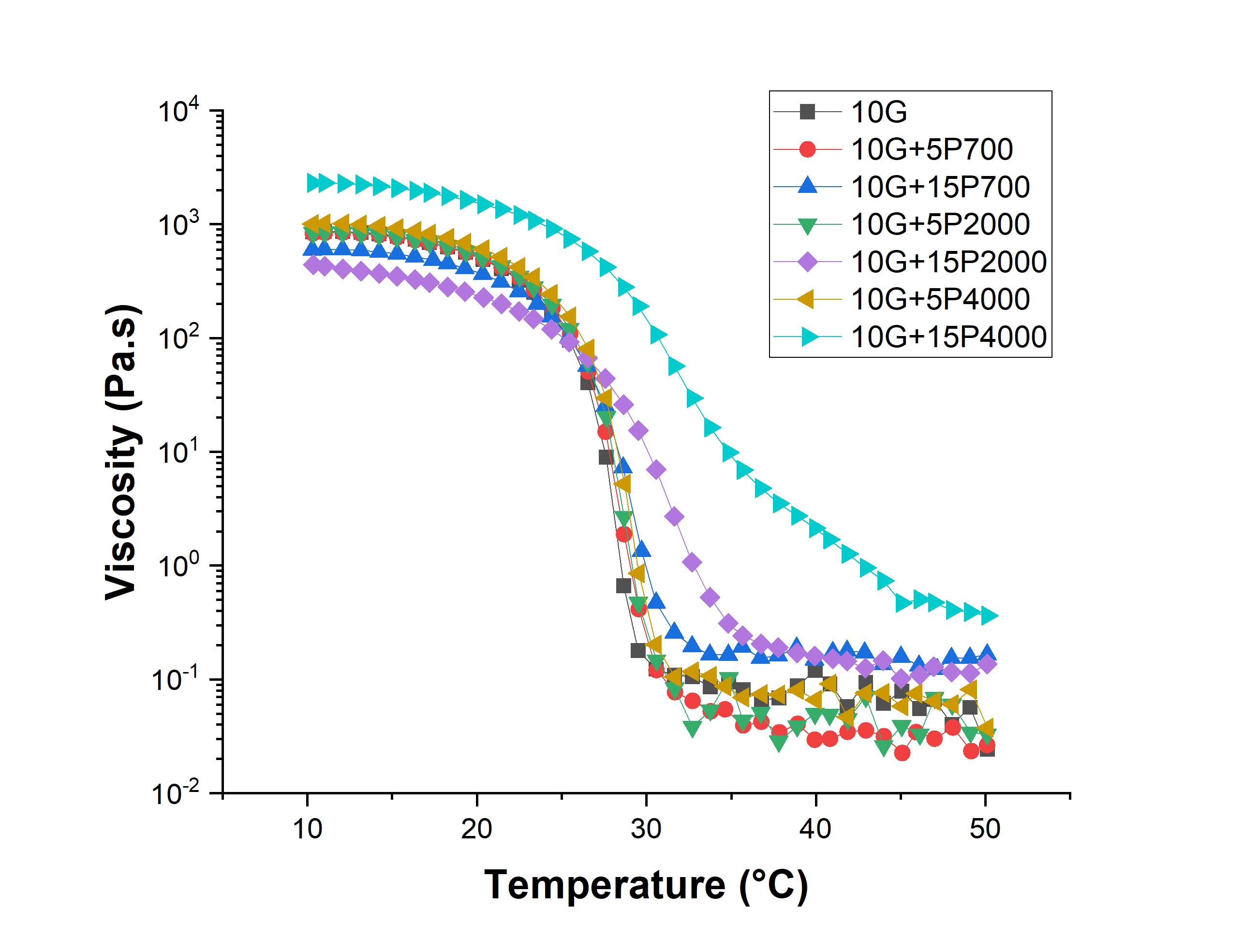


**Figure S2.** Temperature sweep ranging from 10-50°C showing the bioinks viscosity.


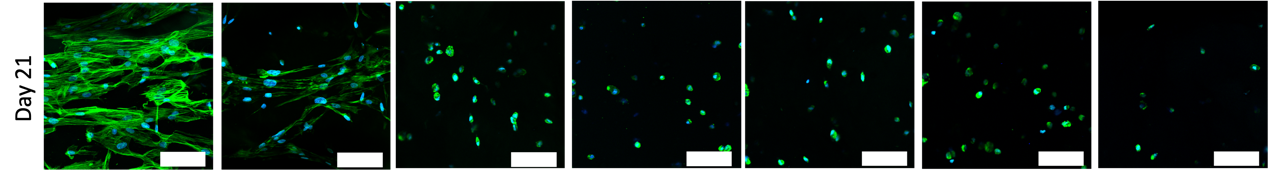
**Figure S3.** Confocal microscope images showing cell morphology in the bioprinted constructs at day 21 (scale = 100 µm; blue=nuclei and green=actin).
